# A Systematic Review on Validated Precision Livestock Farming Technologies for Pig Production and Its Potential to Assess Animal Welfare

**DOI:** 10.3389/fvets.2021.660565

**Published:** 2021-05-14

**Authors:** Yaneth Gómez, Anna H. Stygar, Iris J. M. M. Boumans, Eddie A. M. Bokkers, Lene J. Pedersen, Jarkko K. Niemi, Matti Pastell, Xavier Manteca, Pol Llonch

**Affiliations:** ^1^Department of Animal and Food Science, Universitat Autònoma de Barcelona, Barcelona, Spain; ^2^Bioeconomy and Environment, Natural Resources Institute Finland (Luke), Helsinki, Finland; ^3^Animal Production Systems Group, Wageningen University and Research, Wageningen, Netherlands; ^4^Department of Animal Science, Aarhus University, Tjele, Denmark; ^5^Production Systems, Natural Resources Institute Finland (Luke), Helsinki, Finland

**Keywords:** PLF, sensor, validation, welfare, sows, piglets, fattening pigs

## Abstract

Several precision livestock farming (PLF) technologies, conceived for optimizing farming processes, are developed to detect the physical and behavioral changes of animals continuously and in real-time. The aim of this review was to explore the capacity of existing PLF technologies to contribute to the assessment of pig welfare. In a web search for commercially available PLF for pigs, 83 technologies were identified. A literature search was conducted, following systematic review guidelines (PRISMA), to identify studies on the validation of sensor technologies for assessing animal-based welfare indicators. Two validation levels were defined: internal (evaluation during system building within the same population that were used for system building) and external (evaluation on a different population than during system building). From 2,463 articles found, 111 were selected, which validated some PLF that could be applied to the assessment of animal-based welfare indicators of pigs (7% classified as external, and 93% as internal validation). From our list of commercially available PLF technologies, only 5% had been externally validated. The more often validated technologies were vision-based solutions (*n* = 45), followed by load-cells (*n* = 28; feeders and drinkers, force plates and scales), accelerometers (*n* = 14) and microphones (*n* = 14), thermal cameras (*n* = 10), photoelectric sensors (*n* = 5), radio-frequency identification (RFID) for tracking (*n* = 2), infrared thermometers (*n* = 1), and pyrometer (*n* = 1). Externally validated technologies were photoelectric sensors (*n* = 2), thermal cameras (*n* = 2), microphone (*n* = 1), load-cells (*n* = 1), RFID (*n* = 1), and pyrometer (*n* = 1). Measured traits included activity and posture-related behavior, feeding and drinking, other behavior, physical condition, and health. In conclusion, existing PLF technologies are potential tools for on-farm animal welfare assessment in pig production. However, validation studies are lacking for an important percentage of market available tools, and in particular research and development need to focus on identifying the feature candidates of the measures (e.g., deviations from diurnal pattern, threshold levels) that are valid signals of either negative or positive animal welfare. An important gap identified are the lack of technologies to assess affective states (both positive and negative states).

## Introduction

Animal welfare comprises three components (1): natural living, affective states, and basic health and functioning. Natural living corresponds to the ability of animals to live according to their behavioral needs. An affective state refers to animal's emotions and moods, which can go from negative (e.g., depressed) to positive (e.g., pleasure). Basic health deals with the normal biological functioning and fitness of animals.

These three components of animal welfare can be measured by indicators based, primarily on the animal, but the surrounding environment can also provide useful information. Animal-based indicators provide a more direct measure of the welfare of the animal compared with resource-based indicators. As an example, to assess the absence of prolonged hunger, Welfare Quality® (WQ) (2), one of the most spread animal welfare assessment protocols, uses the body-condition score as an animal-based indicator. However, in the absence of a reliable animal-based indicator for assessing the absence of prolonged thirst, a resource-based indicator such as water supply, is used, which can only inform about an aspect of the environment animals live in.

Knowledge on the welfare of pigs is important for producers (3) and consumers (4). As an example, for producers, poor health or the presence of damaging behavior such as tail biting negatively impact growth performance (5, 6). Diseases and injuries might urge producers to increase the use of antibiotics (7). Regarding consumers, animal welfare is considered as an important aspect of product quality (8), and studies indicate their willingness to pay for pork produced with enhanced welfare (9–11). Goods produced under improved welfare conditions can be communicated to consumers by certification schemes and associated labeling. Most animal welfare labels related to pig farming in Europe have requirements concerning resource-based welfare indicators such as a space allowance, provision of bedding and enrichment, and minimum transportation time (12). However, animal-based indicators have gained more attention, especially after the WQ protocols were published. For example, most pig welfare labels consider mother-offspring interaction through setting a minimum weaning age (e.g., Mehr tierwohl in Germany, Beter Leven in Netherlands, and Bedre Dyrevelfærd in Denmark).

At present, an adequate assessment of farm animal welfare requires a substantial amount of time and effort. Furthermore, current welfare assessment protocols have some other limitations. To mention a few, they do not contain all three components of animal welfare (1), often lack animal-based indicators, focus on expressing the welfare status at group (farm) level instead focusing on the individual (13), and are largely based on human observation (14), which might imply some subjective judgements (15). This means that current protocols provide a limited picture of the welfare of animals throughout their life, restricting the capacity for early detection welfare problems as well as overall life-time welfare.

The use of monitoring technology in animal production systems to optimize farming processes and reduce human workload, often called precision livestock farming (PLF), is growing. According to Berckmans (15), the objective of PLF is to provide the farmers with tools for online and continuous monitoring of the status of the animals and their environment. These tools may therefore help in decision-making and management of the herd (16). Moreover, PLF could contribute with relevant information related to animal welfare in an easier and quicker manner, making continuous welfare assessments more feasible.

Different sensors exist to measure features of individual pig behavior, and/or physical conditions (e.g., accelerometers, microphones, cameras) (17). PLF can add value for the welfare assessment of animals by (1) allowing individual or sub-group tracking, (2) avoiding stressful procedures involving an animal handling during assessment (e.g., by body weight measurements using video cameras instead of manual weighing), and (3) allowing real-time monitoring. In addition, allows implementing early-warning signals of suboptimal status of the animals, to prevent welfare problems (18). PLF technologies have some limitations though. Technologies are created by humans, who set limits for specific problem detection (e.g., tail biting), so could also be burdened with certain subjectivity (18, 19). Also, as demonstrated in large-case studies for sensor profitability in dairy farmers, investment in PLF technologies might not necessary lead to economic gain (20, 21). In addition, not all PLF tools have an automatic alert, making a gap between the time of problem detection and the potential intervention of the staff. Reliability of data management could be considered a further limitation, since it is carried out by the PLF manufacturing company, which in fact are the data owners. To improve transparency, evaluation on the PLF tools performance by external bodies is essential.

A procedure for validation in the real operation environment of a technology is required before it is transferred to the market (22). Validation is the procedure for evaluating the performance of a technology contrasted with a gold standard to know if it achieves satisfactory prediction accuracy of a measured trait (23). For instance, how well a thermal camera detects fever, compared with a standard thermometer, or how well an automatic feeding system can detect feeding behavior. This validation procedure should be performed internally (on a sample of individuals during the system building), but also externally (on different individuals than those used during building phase) (24). For the sake of transparency, buyers (i.e., farmers) need to know the exact features of the technology they are buying and how accurate they monitor a given condition. It is preferable that the external validation need is carried out by independent bodies.

To the best of our knowledge, an overview of existing PLF technologies that potentially can be used for pig welfare assessments and the validity and reliability of these technologies, is still lacking. The aim of this review is to explore market available PLF technologies that are potentially applicable in commercial pig production, and to review (1) their ability to contribute to longitudinal welfare assessment, and (2) their state of validation. This review focus on technologies that have been validated (either internal or external) and which results have been published.

## Materials and Methods

### Search for Commercially Available Technologies

A web search to identify commercially available PLF systems for pigs was conducted by using Google search engine by one researcher (YG), between February and April 2020. Search terms included pigs (and related words such as sows, piglets), and different technologies known to monitor animal-based welfare indicators for pigs. Technologies provided by a wide range of suppliers were scanned. More specifically, the search criteria included the following animal categories: *(pig), (piglet), (weaner), (fattener pig), (sow)*, and the technology using one of the following terms: *(automatic drinker OR automatic waterer), (automatic feeder), (electronic feeding station), (activity sensor OR activity monitor), (RFID), (GPS), (thermal camera), (infrared thermometer), (automatic weigh scale), (sorting scale), (weight camera), (body condition score sensor OR automatic body condition score), (body condition camera), (lameness sensor), (automatic lameness detection), (pressure mat OR force sensor), (automatic behavior analyzer), (image-based behavior analyzer), (body-temperature sensor), (automatic sound analysis), (cough sensor OR cough monitor)*. No boolean operators were applied, except OR boolean, as Google does not allow the use of ^*^ to automatically fill the search term to include related words. The example search looked as follows: *pig automatic weigh scale OR automatic weigher*.

The first five pages (50 hits) of results in each search were reviewed. Only commercially available technologies were selected for further review, excluding prototypes or devices in the building phase. If required, technology providers were approached to clarify the stage of development. Information on a sensor name, provider name, internet link, sensor type, aim, and provider country were summarized. Information regarding the production phase that the technology is applicable or designed for, was also specified.

### Literature Search

Following the Preferred Reporting Items for Systematic Reviews and Meta-Analysis (PRISMA) guidelines (25), a literature search was conducted by one researcher (YG), and verified by a second researcher (AS). The search was focused on finding external validation studies on PLF technologies for pig welfare. In addition, the obtained data set (studies reporting different validation levels) was used for checking internal validation to find potential technologies for pig welfare monitoring that are not yet externally validated.

The literature search was conducted through Web of Science and Scopus databases, between the 1st of June and the 31st of July 2020. Search terms included: different phases in the production cycle of pigs, terms regarding validation, types of sensor or their commercial names. Besides, some animal-based welfare indicators were included as search terms, including body temperature, body weight, and locomotion as physical condition indicators; activity, feeding, drinking and vocalizations as behavioral indicators; and cough and lameness as physiological indicators. Search terms related to individual recognition and animal location in the pen were also included.

Search terms were applied to title, abstract and keywords as follows:

(pig OR sow OR weaner OR piglet OR fattenn^*^)

AND

(validat^*^ OR evaluat^*^ OR assess^*^ OR test^*^)

AND (one of the following search combinations)

1. (accelerometer), ((“activity sensor” OR “motion sensor” OR “locomotion sensor” OR “infrared motion” OR (activity AND automat^*^))
2. ((position^*^ *AND sensor) OR rfid OR “tracking system”)*
3. *((vision AND camera) OR “image analysis”)*
4. ((thermistor OR infrared) OR (body temperature) AND (monitor^*^ OR detect^*^ *OR sensor)*
5. ((scale^*^ AND weigh^*^) AND automat^*^)
6. (“body condition scor^*^” AND sensor OR automat^*^)
7. ((“feeding behavior^*^” OR “feeding behavior^*^”*) AND sensor)*
8. (“feeding station” OR “feed^*^ *meter” OR “water meter” OR “automatic feeder”)*
9. (“drinking behavio^*^” *AND monitoring)*
10. ((sound AND sensor) OR (cough AND detect^*^))
11. *(respiratory AND distress AND monitor)*
12. ((sound AND sensor) OR (vocali^*^ AND detect^*^))
13. ((gait OR lameness OR lame^*^) AND (sensor OR “image analy^*^” OR image OR automat^*^ OR mat OR “pressure mat” OR “pressure sensor” OR “force plate^*^”*))*

NOT (review OR beef OR sheep OR survey OR goat^*^ OR hors^*^ OR pipeline OR genom^*^ *OR “wild boar” OR “swine model” OR “porcine model”*).

To make sure that all technologies identified in the first search were checked for validation, an additional search of literature using the name of identified commercial sensors in Google (Supplementary Table 1) was performed. An example of search criteria for “FLIR T300” technology was: *pig OR piglet OR weaner OR fattener OR sow FLIR T300*.

### Inclusion and Exclusion Criteria

Only peer-reviewed articles, written in English and published between January 2000 and July 2020 were considered. Articles related to welfare assessment in species other than domesticated pigs (*Sus scrofa*) were excluded. Studies not addressing technology development or validation, as well as studies using a PLF technology, but not testing its performance or validating it, were also excluded.

Only articles addressing automated and on-farm applicable PLF technologies were included in this review. Studies testing on pigs not meant for farm practices (e.g., minipigs) were excluded. Articles neither dealing with aspects directly related to animal welfare (such as estrus detection) nor with animal-based welfare indicators (e.g., environmental measurements such as climatic aspects) were excluded. Duplicates were also removed from the data set.

Selected studies were grouped based on the type of PLF technology [accelerometers, photoelectric sensors, RFID (Radio Frequency Identification), load-cells, flow meters, microphones, cameras, thermal cameras, infrared (IR) thermometers, pyrometers]. The final data set included sample size, production phase, and the relevant animal-based indicator(s).

### Study Classification

A gold standard is defined as a criterium by which given tool was evaluated (26, 27). In the conducted review, there were three possible options:

1. tool was validated against a human observer,
2. tool was validated against other tool with well-defined performance record,
3. tool was validated based on its ability to detect change in animal behavior or physical condition during planned experiment.

As in Stygar et al. (28), a similar review but focusing on dairy cattle PLF technologies to monitor animal welfare, and based on Altman et al. (24), we defined the following levels of validation:

1. External self-validation: studies where the system was evaluated using a fully independent data set, meaning that data was collected from different herds not used for system development. Validation was conducted by either one scientist, at least, involved in the technology development or by someone representing the company who owns the technology.
2. External independent validation: studies where the technology was validated using a fully independent data set, from different herds than those used for technology development, and research was conducted by independent scientists with no relationship with the company that owns the technology.
3. Internal validation: studies where the technology was validated using the same data set as for technology building, or where the commercial name of the technology was not specified, or the origin of the validation data set was unknown.

For determining the validation level within the literature search, the technology and the validation location were identified. The technologies were identified by looking for their commercial names or papers describing its development phase (prototypes). Studies where the specific location of herd was not mentioned (for example due to privacy concerns), but clearly used different herds than for system building, were included as external validation level.

## Results

### Commercially Available Technologies

**Figure 1:**
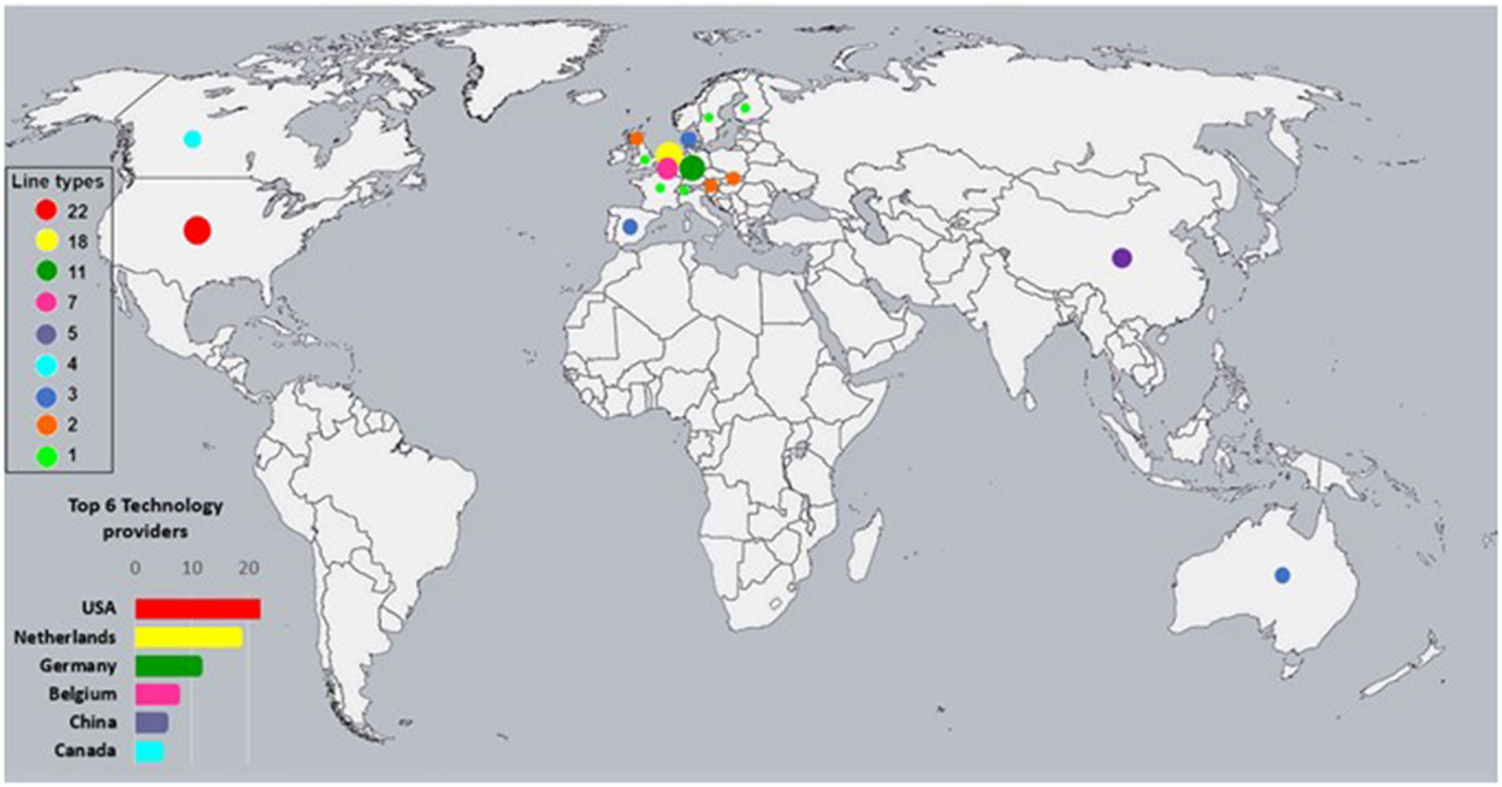
Countries of origin of commercially available PLF technologies with potential use in pig welfare assessment. For companies with multiple locations, address of the headquarter was used. Some companies have operations in more than one country.

All PLF technologies with a potential link to animal-based pig welfare assessment are listed in the Supplementary Table 1. In total 83 technologies were found, based on 10 different types of sensors, from 46 different providers whose headquarters are located in 17 countries. Figure 1 shows the origin of the commercially available technologies. Most of the providers are located in the United States of America (*n* = 22), the Netherlands (*n* = 18), and Germany (*n* = 11), followed by Belgium (*n* = 7), China (*n* = 5), and Canada (*n* = 4). Location of providers was identified in a minor extent in other countries (including Spain, Australia, Slovakia, Scotland, Austria, Switzerland, Turkey, Sweden and England).

As summarized in Table 1, load-cells based and vision-based technologies were the largest groups of identified technologies. Thermal-image technology was the third most common type of sensor. Remaining identified technologies included microphones, accelerometers, body temperature devices, photoelectric sensors, GPS (Global Positioning System), and RFID (for animal tracking). Most of the identified commercial tools can be used for different pig production phases, however, some are targeted at a specific production phase. Of the commercially available technologies, 39% was used for fattening pigs, 33% for sows, and 28% for piglets and weaned piglets. Load-cells based and vision-based body-weight tools are mainly used for fattening pigs. No technologies exclusively developed or adjusted for piglets and weaners were found.

**Table 1:** Commercially available Precision Livestock Farming technologies categorized by the sensor type and measured trait.

**Type of technology**		**Animal-based measure**	**Number of identified technologies**		**% over total commercial solutions (*****n*** **= 83)**	
Load cells and flow meters	Force plates	Gait attributes	2	Load cells with RFID 18	22%	45%
	Load cells	Feed intake	3			
	Flow meter	Water intake	2			
	Load cells/Flow meter		1			
	Feeder/drinker	Feed/water intake	5			
	Scale	Body weight	5			
	Feeder/drinker/RFID	Feed/water intake/body weight	15	Load cells without RFID 19	23%	
	Scale/RFID	Body weight	4			
Cameras		Body weight	14	22	26%	
		Behavior and activity	8			
Thermal cameras		Body temperature	10		12%	
Microphones		Cough	2	5	6%	
		Animals sounds	3			
Accelerometers		Activity	4		5%	
Body temperature devices	Contact-temperature device	Body temperature	2		2%	
	Pyrometer	Body temperature				
Photoelectric sensors		Lameness	2		2%	
GPS		Location	1		1%	
RFID		Individual identification and tracking	1		1%	

### Literature Search on Validation Trials

**Figure 2:**
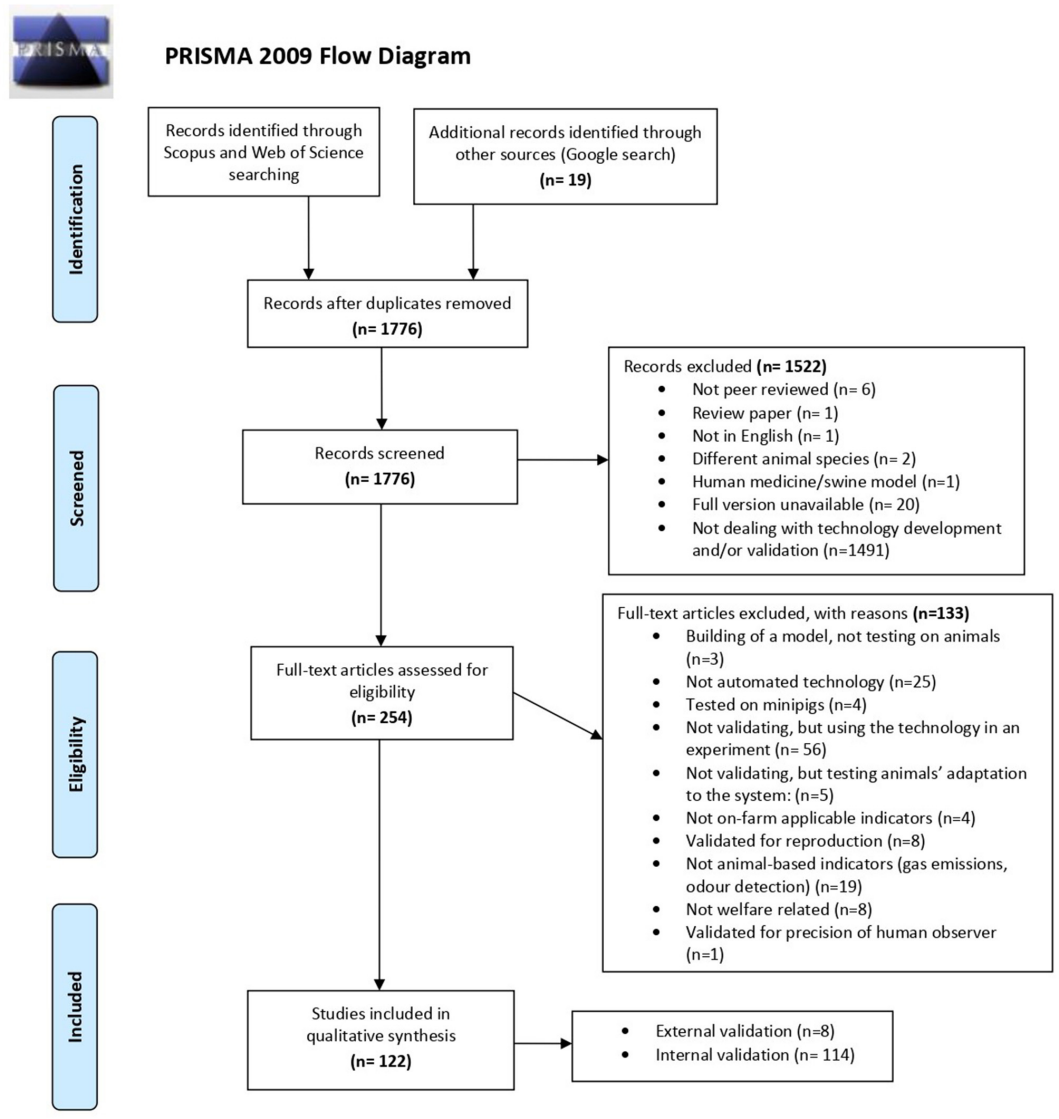
Modified PRISMA flow diagram (25) with the systematic review search strategy and study selection.

The literature search through databases provided 2,463 results. Nineteen studies used the commercial names of technologies identified in the web search. After removing duplicates and applying the inclusion and exclusion criteria, 111 studies remained. The PRISMA flow diagram in Figure 2 describes the stages of studies selection process and reasons for exclusion.

**Figure 3:**
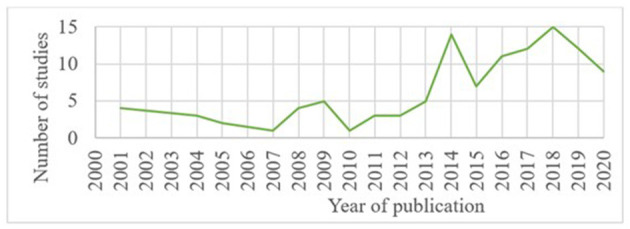
Temporal distribution of validation studies on PLF technologies included in this review, with potential use in pig welfare assessment.

**Figure 4:**
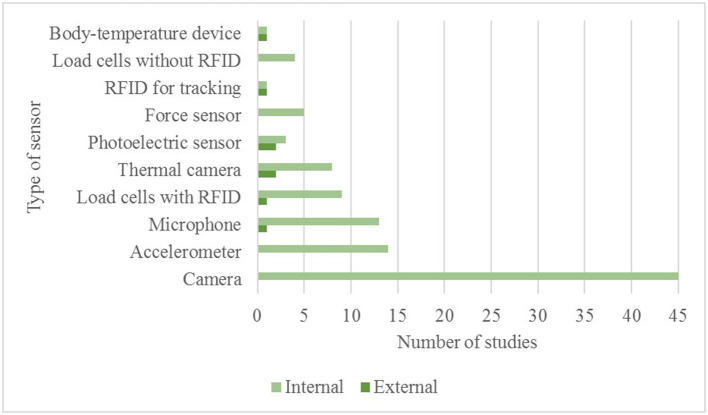
Number of studies classified as internal or external validation for different sensor categories.

As illustrated in Figure 3, the number of publications on PLF internal and external validation increased over the last decade. Neither the internal nor the external validation studies followed any particular pattern of temporal distribution of the publications. Only eight (7%) of the 111 selected studies, fulfilled the external validation criteria, whereas 103 (93%) were classified as having an internal validation (Figure 4). Within the internal validation studies, 23 (22%), did not meet the criteria for external validation, but could be included as internal validation. In 18 of those 23 studies, the name or origin of the sensor was not provided; it was therefore impossible to identify its commercial availability or development stage. This applied to nine studies with camera-based technologies (29–37), three studies on load-cells [a drinker (38), a scale (39), and a force plate (40)], two on RFID (41, 42), two on accelerometer (including one on accelerometer and microchip for body-temperature) (43, 44), one study on microphone (45), and one on load cells with RFID (46). In the other five of those 23 studies, the origin or location of the herds used, or the origin of the sensor was not described [two studies on thermal cameras (47, 48), one on load cells with RFID in a feeding station (49), one on cameras (50), and one on microphone (51)]. From the obtained list of commercially available PLF technologies, 14% were validated in some identified papers of literature search (12 of 83 technologies), of which 5% corresponded to external validation (52–55).

An overview of internal and external validation studies can be found in Table 2. Most internal validation studies concerned camera-based technologies, followed by load-cells based technologies. The next most frequent validated type of sensors were accelerometers and microphones, followed by thermal-cameras, photoelectric sensors, flow meters, and RFID (for animal tracking). The less common validated technologies were non-contact body-temperature sensors (infrared thermometers, and pyrometer). All validation studies, together with performance indicators, are described in detail in Supplementary Table 2.

**Table 2:** Number of peer-reviewed validation studies on sensor technologies used in pig production, categorized by sensor type and validation level (internal or external).

**Type of sensor**	**Number of internal validation studies**	**Number of external validation studies**	**Total number of validation studies**
Camera	45	0	**45**
Load-cells	With RFID- 8 (Feeders-9 Drinker-1)	With RFID- 1 (Feeder-1)	With RFID- **10** (Feeders-10 Drinker-1)
	Without RFID- 7 (Force plates-5 Scales-2)	Without RFID- 0	Without RFID- **7** (Force plates-5 Scales-2)
Accelerometer	14	0	**14**
Microphone	13	1	**14**
Thermal camera	8	2	**10**
Photoelectric sensors	3	2	**5**
Flow meters	2	0	**2**
RFID	1	1	**2**
Non-contact body-temperature sensors	Infrared thermometer- 1	Pyrometer- 1	Infrared thermometer- **1** Pyrometer- **1**

*The bold numbers indicates the total sum of the number of internal and external validation studies on each type of sensor. In brackets: the specific sensors included in each sensor type category*.

**Figure 5:**
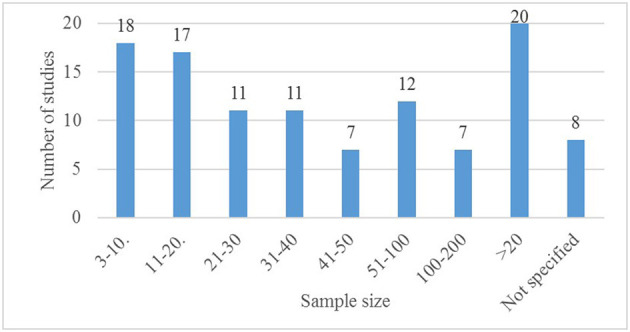
Sample size (the number of animals) used for external or internal validation in the reviewed studies.

Regarding the productive phase of animals used for the studies, the most frequently used pigs were fatteners (51 studies), followed by sows (28 studies), and weaners (21 studies). Sensors for piglets and gilts were less frequent (eight and five studies, respectively). In our results on commercial search, no PLF solution developed or adapted exclusively for piglets or weaners was identified. However, research on PLF solutions for piglets exists, as studies on cameras, thermal cameras, feeders with RFID, microphones, photoelectric sensors, pyrometers and RFID for tracking were identified using young pigs (from birth to 10 weeks old or up to 70 days old) as target animals. Five studies used pigs in general, not specifying the productive phase. Sample size used in the selected studies are illustrated in Figure 5. Some patterns were observed in relation to the size of the samples and validated technology. The smallest sample size (including samples of <10 animals) was used in studies validating cameras (eight studies), accelerometers (five studies), microphones (three studies), RFID for tracking (one study), and force plates (one study). However, most of the studies on accelerometers (11 out of 14 studies) and force plates (four out of five studies) were conducted using sample sizes smaller than 24 animals. Automatic feeders and drinkers, with or without RFID, and sorting scales systems, were mostly validated in studies using sample sizes from 55 to more than 1,000 animals. Three studies on drinkers with RFID validated the technology using samples between 25 and 30 animals. Studies validating accelerometers, force plates, cameras, microphones and RFID for tracking of animals used samples sizes from 3 to more than 500 animals. Studies on thermal cameras and photoelectric sensors also were performed using varied ranges of sample sizes (from 11 to 297 animals). In the external validation studies sample sizes were between 20 and 63 animals.

### Validation Studies and Technologies for Welfare Assessment in Pigs

#### External Validation Studies

Table 3 summarizes the externally validated (self-validation and independent validation) technologies with potential use for pig welfare assessment.

**Table 3:** Studies on externally validated (independent or self-validated) sensor technologies with potential use in pig welfare assessment, specifying the sensor type, commercial name, the animal-based indicator assessed and its evaluation level (individual or group).

**Technology name**	**Indicator**	**Reason of use (monitored trait)**	**Evaluation level**	**Nr of validation trials**	**Used sensors**	**Independent validation^a^**	**Self-validation^b^**
OPTEX RX-40QZ	Activity and posture-related behavior	Active and/or passive (without distinguishing on activity type)	Group	1	Photoelectric	(56)	
STREMODO (commercially unavailable)	Physical condition	Stress vocalization (due to handling)	Group	1	Microphone		(57)
FLIR E5 thermal imaging camera	Physical condition	Body temperature	Individual	2	Thermal camera	(53)	
FLIR ThermoCAM S60	Physical condition	Body temperature	Individual			(54)	
FIRE	Physical condition	Body weight	Individual	1	Load cells and RFID	(52)	
	Feeding and drinking behavior	Feed intake (kg)					
Pyrometer Optris	Physical condition	Body temperature	Individual	1	Pyrometer	(55)	
Prototype system	Feeding and drinking behavior	Feeding behavior, feeding time and/frequency	Individual	1	RFID		(58)
Standing lying sensor	Activity and posture-related behavior	Posture change (between lying, standing and sitting)	Individual	1	Photoelectric	(59)	

a *External independent validation—validated using independent data set (different animals and herd than for technology building) and co-authors were not involved in technology development*.

b *External self-validation—validated using independent data set (different animals and herd than for technology building) and was developed and validated by at least one the same co-author (based on the authorship of papers) or have been validated by at least one co-author representing a company providing a technology*.

#### Measured Traits and Technologies in Internal and External Validation Studies

Table 4 provides an overview of technologies tested to monitor different welfare indicators related to pig production. Validated traits were grouped in following categories: activity and posture-related behavior, feeding and drinking behavior, other behaviors, physical condition, and health-related traits.

**Table 4:** Summary of internally and externally validated technologies to monitor different pig welfare indicators, classified by monitored trait and sensor type.

**Indicator**	**Reason of use (monitored trait)**	**Technologies tested**
Activity and posture-related behavior	Active and/or passive (without distinguishing on activity type)	Accelerometer (60–65) Photoelectric sensor (56)^a^, (66–68) Camera (30, 69–71)
	Lying	Camera (72–76) Accelerometer (43, 60–62, 77, 78)
	Standing	Camera (72–74, 79) Accelerometer (43, 62, 77)
	Sitting	Camera (72–74) Accelerometer (43, 77)
	Kneeling	Camera (73, 74)
	Posture state and transitions between states (e.g., between lying and standing)	Photoelectric sensor (59)^a^ Accelerometer (78) Camera (74)
	General motion activity and tracking (related to thermal comfort)	Camera (34, 76, 80) Accelerometer (78) Thermal camera (81)
	Walking (number of steps)	Accelerometer (61)
	Tracking (identifying location or number of animals)	Camera (32, 82–84) RFID (41)
Feeding and drinking behavior	Feed intake (kg)	Load cells with RFID (52)^a^, (49, 85)
	Feeding time and/or frequency	RFID (42, 58, 86)^a^, (87, 88) RFID and environment temperature and humidity sensors (46) Camera (37, 73, 74) Accelerometer (61, 64)
	Hunger stress identification	Thermal camera (89) Microphone (90–92)
	Nursing, suckling	Camera (37)
	Drinking time and/or frequency	RFID (93) Accelerometer (64) Camera (37, 73, 74, 94–97)
	Thirst stress identification	Thermal camera (89) Microphone (90, 92)
Other behavior	Nest- building behavior	Accelerometer (98)
	Aggressive behavior	Camera (99–104) Accelerometer (64)
	Cascade defense (freezing and startle duration)	Camera (105)
	Rooting	Accelerometer (61)
	Mounting behavior	Camera (97, 106)
	Tail biting	Camera (50, 107) Water flow meter and environment temperature sensor (108)
	Exploratory behavior	Accelerometer (64)
	Playing behavior	Camera (96)^b^ Accelerometer (64)^c^
Physical condition	Gait attributes	Load cells [force plates, (35, 40, 109–111)] Camera and accelerometer (112), Camera (113)
	Cough detection	Microphone (45, 51, 114, 115)
	Body weight	Camera (29, 33, 36, 116–122) Load cells (scales) with RFID (52)^a^ Load cells (scales) (39, 123)
	Muscle score	Camera (124)
	Body temperature	Thermal camera (48, 53)^a^, (54)^a^, (125, 126) Pyrometer (55)^a^
	Stress (e.g., due to heat or cold, pain, fear)	Microphone (57, 90)^a^, (91, 92, 127–130) Thermal camera (89)
Health-related traits	Lameness and claw lesions detection	Accelerometer (131) Camera (35) Thermal camera (132)
	African Swine Fever (sign: changes in activity level)	Camera (31) Accelerometer and microchip for body temperature (44)
	Influenza A virus (signs: fever) and changes in activity level	IR thermometer (133)
	Respiratory disease	Thermal camera (47, 134) Microphone (135, 136)
	General health problems	RFID (137)
	Diarrhea	Water flow meter (38)

a *External validation study*.

b *Water base play*.

c *Use of manipulating material*.

#### Activity and Posture-Related Behavior

We identified five sensor types (cameras, accelerometers, photoelectric sensors, thermal cameras, and RFID) that were used for activity measurement (Table 4). The following traits were monitored: general motion activity (active, inactive state), walking (number of steps, identified as separate behavior), tracking (identifying location or number of animals in this location), postural state and transition between states (lying, standing and sitting), as well as general motion activity and tracking (studied in relation to thermal comfort). We identified five sensor types (cameras, accelerometers, photoelectric sensors, thermal cameras, and RFID) that were used for activity measurement. Studies on accelerometers were mostly developed for sows, to classify postures and activity. Several studies validating the use of image analysis for postural states monitoring were found. For activity traits related to tracking (individual recognition and pen location), two types of sensors were identified: cameras and RFID. General motion activity and tracking related to thermal comfort (clustering behavior) was assessed using thermal-imaging.

#### Feeding and Drinking Behavior

Five types of technologies were identified for monitoring feeding and drinking behavior: RFID (feeders and drinkers), cameras, accelerometers, thermal cameras, and microphones (Table 4). Measured traits were: feed intake, feeding and drinking frequency and duration, stress related to hunger or thirst, as well as nursing and suckling behavior. Sows' nursing behavior was monitored using cameras (37). The estimation of stress conditions related to hunger and thirst was assessed by vocalizations (90–92) using microphones and via skin temperature using thermal cameras (89), applying different stressors to the animals. Feed intake was monitored using RFID in an electronic feeding station (52, 85). Evidence suggests that the performance of RFID feeders for monitoring feeding behavior is negatively affected by accumulation of debris under the feed trough, the large number of pigs per feeder space and pen space allowance (52). Therefore, frequent recalibration of the device is needed. Other studies validated feeding stations for monitoring individual daily feed intake (49). RFID systems were also validated for registering feeding and drinking patterns of individual growing-finishing pigs (58, 86, 93). Drinking patterns can be monitored using video analysis for evaluating visits to the drinker and contact time (94, 95), and for distinguishing drinking from drinker-playing behavior (96). Cameras for the identification of behavior of sows were used for identifying feeding and drinking behavior in the farrowing crate (37, 73, 74), as well as in group-housed sows (97).

#### Other Behavior

For monitoring other behavior, accelerometers, cameras, and water flow meters were used (Table 4). Cameras were the most often tested for monitoring other behavior (*n* = 12), followed by accelerometers (*n* = 5). Cameras were used for assessing behavior as a predictor of tail biting outbreak (restlessness) (107), as well as for recognizing high and medium aggression events based on image detection of motion and acceleration (as displacement in image) (100–103). Accelerometers were used for assessing movement associated to nest-building (98) and aggression (64). Water flow meters have been used in a study for predicting tail biting outbreaks by combining the frequency of use of water points and ambient temperature (108). Image analysis was also used for recognizing movement and location associated to walking, running, exploring, playing, nursing, feeding, urinating and mounting. Image methods for analyzing low tail posture as an early warning of tail biting have been studied (50). None of the vision-based tools have been externally validated (see Table 2).

#### Physical Condition

The following technologies were identified for monitoring physical condition: load cells (force plates, scales), load cells with RFID, cameras, microphones, thermal cameras, and pyrometer (Table 4). Measured traits included gait attributes (weight distribution on legs, gait characteristics, axial body movements trajectory during walking), cough, body temperature, stress (e.g., due to heat or cold, pain, fear), body weight as well as muscle score (loss in muscle condition is associated to acute and chronic diseases, and affects strength, immune function, and wound healing). Body weight was the most studied attribute, followed by stress and gait characteristics. Cameras were frequently used to assess body weight. One study tested the potential of depth-image analysis to evaluate axial body movements trajectory during walking, as an early indicator of lameness (113). Microphones were applied for evaluating the features of stress vocalizations, applying stressors such as handling, cold, heat, pain, hunger and thirst (eight studies) and for cough detection. Load cells (force plates) were applied for gait characteristics assessment. Thermal image was used for assessing body-temperature as an alternative of rectal temperature measurement. Also, the usefulness of thermal image to assess piglets' stress by measuring body-temperature changes when applying stressors (cold, pain, hunger, thirst) was tested (89). One study was found using a pyrometer for continuously measuring body-temperature, showing negative validation results (55). Load cells (scales) with and without RFID were validated for assessing body weight.

#### Health-Related Traits

Seven technologies were identified for assessing health-related traits: cameras, accelerometers, infrared thermometer, thermal cameras, microphones, RFID, and water flow meters (Table 4). The following health-related traits were assessed: lameness, claw lesions, detection of signs of disease associated to African Swine fever (decrease in activity), as well as Influenza A virus (fever), respiratory disease, diarrhea, and general health problems. Respiratory disease was the most frequent studied health-related trait (four studies), followed by body-temperature to detect fever (three studies), and lameness (three studies). Acceleration in combination with body-temperature data was tested for generating early alerts of disease (44). Acceleration was also applied for lameness detection based on sows' postures (131). Thermal imaging for assessing health problems was applied in three studies: one for detecting inflammation related to lameness in pregnant sows (132), and two for respiratory disease assessment (measuring skin-temperature at chest level for detecting lung tissue damage) (47, 134). One study tested the use of infrared thermometry for fever detection (133). Microphones for cough detection to identify sick pigs was applied in two studies. Moreover, RFID data were used to detect deviations in individual pigs' feeding patterns to point diseases or other disturbances, correlating it with the Welfare Quality® protocol assessment (looking for skin, ear and tail lesions, soiling, abnormalities in body condition, respiration, locomotion, bursitis, lameness, or diarrhea) (137). Finally, water usage data from flow meters have been tested as an early indicator of potential presence of diseases at group level, demonstrating that changes in diurnal drinking patterns of pigs can predict, for example, a diarrhea outbreak before clinical signs show up (38).

## Discussion

The aim of this review was to explore existing PLF technologies that potentially can contribute to measure animal-based welfare indicators of pigs and investigate their validation status. There is a substantial number of PLF tools (83 in our commercial list) in the market that can be potentially used to assess animal-based indicators of pig welfare. However, only a limited number of technologies have been internally validated, and only four market available technologies were externally validated (two thermal cameras, one pyrometer for monitoring body-temperature, and one RFID feeding station for monitoring feed intake and body weight) (52–55). Through this review, we identified important gaps in terms of validation on commercially available sensors. PLF tools that can identify stress due to hunger and thirst (90–92) have been found in the literature search but not in the commercial search. Similarly, tools that can assess play (64, 96), exploratory behavior (64), and aggressive behavior (64, 99–104) as well as models trained for recognizing specific diseases, such as African swine fever (31, 44), are not yet commercially available. The combination of different sensors as part of the same PLF solution was identified in several studies of the literature search, but they were not found as commercial solutions in our list (except for the combination of RFID and load cells, and accelerometers with body temperature sensors attached to ear tags).

Initially, we were searching for externally validated tools. However, only eight tools with external validation records were found. Therefore, the obtained data set has been used to find out which technologies have potential to contribute to pig welfare monitoring, but are not yet externally validated. Among the market available PLF tools, only 14% were found in validation studies. However, it needs to be noted that information obtained from the market is an overview of available PFL tools, as only products with websites and commercial information in English were included. Besides, several solutions may have been left out of the list, as we excluded technologies not addressing animal-based welfare indicators, or without direct involvement with animal welfare, for instance, those measuring reproductive parameters [e.g., (138–141)], or animal identification, such as facial recognition (142, 143).

Also, the literature search on validation studies may not have included all relevant PLF technologies for measuring animal-based welfare indicators. The reason for this was the choice of search criteria. Our search criteria specified the type of sensor applied to title, abstract and keywords. For this reason, some studies which mention sensor type only in material and methods section were omitted. This was the case for one study on image-analysis, one on water meters and one on load-cells (144–146) for instance. Pen fouling outbreaks, which can cause health problems due to poor hygiene, can be predicted by analyzing lying behavior using machine learning (144) and drinking patterns from water meters (146). The usefulness of load-cells to detect abnormalities in growth patterns of pigs at group level has been proved, even if the animals are not individually identified, by measuring the initial body weight, average daily gain and daily fluctuations in body weight parameters (145). One of the exclusion criteria was to remove articles not dealing with animal-based welfare indicators. Hence, all papers that use environmental data for welfare monitoring [e.g., (147), using ambient temperature data] were excluded.

The recent development of certain technologies, such as computer vision based technologies (analysis of static images and video, 2D, 3D and thermal-imaging) begin to appear on the market (148). In our review, vision-based PLF was the type of technology that could have potentially assessed the largest number of animal-based welfare indicators. However, most studies using computer vision for monitoring measures related to animal welfare assessment still report some need of improvement. For instance, in automatic body-weight detection, there is a need for development of algorithms accounting for the effect of gender and genotype (118), and the refinement of algorithms on automatic detection rate of pig boundaries (116). Similarly, for lameness detection, some reports have suggested the need for algorithms refinement to increase sensitivity and reliability (113), and the need to incorporate additional elements to the system, such as infrared lights (35). None of the reviewed systems were externally validated.

### Performance of Validated and Commercially Available PLF Technologies, and Its Potential for Pig Welfare Assessment

According to our results there are no guidelines on the reporting of performance information in PLF validation studies. For that reason, the differences in performance measures reported by validation studies were not used as exclusion criteria. To be considered valid and feasible in commercial conditions, the performance of a technology should be tested in multiple practical scenarios, in different types of production systems and with different housing environments. In the reviewed studies, external validation was only performed in 7% of studies. Low number of validation studies can be explained by: (i) insufficient reporting (e.g., lack of information on validation place), (ii) low scientific interest (e.g., reluctance of scientific journals to publish validation studies on tool not applied for research), (iii) high costs and labor intensity of data collection, (iv) reluctance to publish negative results, and (v) the recent development of certain technologies. According to our results, validation trials for commercial purposes were less common than for research purposes, and it could be due to time and resources requirements for validation. Besides, market available PLF technologies for pigs are mostly calibrated by the providers, and its precision and reliability on data management is assumed by them without an independent validation (Table 3). The fact that PLF companies perform validation trials themselves, and could obtain negative results without reporting these, has to be considered as an important reason for reluctance of dissemination.

Concerning the quality of reporting, external validation requires specific information on the location of the trials, the name of validated device, software provider, and studied population, knowledge about the origin of the animals, if the test procedure was applied in commercial or experimental conditions, and clear information on which golden standard was used for validation and how it was measured. Information gaps in reporting were found in 22% of studies, for which reason were classified as internal validation studies. Few examples are the study of Petry et al. (48), and Guarino et al. (51), which despite reporting their results under laboratory and practical conditions, presented lacks of information in materials and methods (regarding used animals, and study location, respectively).

In addition, internal validation studies with samples smaller than 10 or 20 animals were very frequent (validating some cameras, accelerometers, microphones, RFID for tracking, and force plates). It was observed that larger samples (above 20 animals) were mainly used in studies validating feeders and drinkers with or without RFID, and sorting scales. According to Royston and Altman (23), an appropriate validation sample is required to provide a reasonably accurate estimate of a measure and to avoid the risk of false negatives. Thus, studies with limited sample sizes could have low validity and are inconclusive (23). However, at present, a standardized parameter is not known for what could be considered a reasonable sample size (depending on the type of technology to be validated). A remarkable lack was found regarding technologies developed or with adapted algorithms for young pigs exclusively. Thus, there is an important concern in regard to the usefulness of PLF for monitoring the welfare of young animals.

As stated by Stygar et al. (28), in the case of the dairy cow industry, devices used for the official recording of milk (such as milk samplers) must comply with the requirements of the International Organization for Standardization (ISO) to obtain the certification, and must be tested for approval by the International Committee for Animal Recording and Analysis (ICAR) (149). Recommendations on proper validation procedures for PLF technologies for pig industry are still lacking.

There is a constant development of PLF technologies to offer solutions for animal production including animal welfare. Despite the lack of external validation for the majority of technologies, the link between the feature measured by a sensor and the state of the animal in terms of welfare is not always clear. For instance, camera-based motion detection is often mentioned as a tool for welfare assessment. However, few studies have demonstrated a clear link between features of motion and specific animal welfare problems, such as lameness (113), or specific diseases (31). The performance of identified types of PLF for monitoring animal-based welfare indicators and measured traits in validation studies will be described below. The types of sensors are listed in descending order, according to the number of validation studies compiled for each. Supplementary Table 2 shows full information on the validation results of each study.

#### Camera-Based Technologies

Internal validation of the vision-based technologies in many cases reported very promising results with accuracy above 95% [e.g., (30, 33, 58, 73, 74, 93, 95, 97, 107, 145–147)]. However, none of the outstanding performance results for vision-based monitoring have been confirmed by external validation. Image-analysis has been used for assessing sows' postures, such as standing, lying, and sitting (73), evaluating lying patterns in group-housed pigs responding to thermal conditions of the pen (76, 80), detecting animals' location (32, 84), distinguishing drinking and drinker-playing behavior (96), identifying feeding behavior (37, 73, 74, 97), recognizing aggression events (100–103) and tail biting (50), estimating body weight (36, 118, 119), and detecting African swine fever (31).

Changes in animals' postures can be used as health indicators (31). Although the assessment of certain postures (sitting, kneeling) is not very accurate using vision based technologies (72), it is possible to distinguish standing active behaviors, such as feeding or walking, against resting patterns (30, 69). Lying posture, predicted by image-analysis, can indicate health problems. For instance, resting duration and frequency changes due to diseases (31), lesions and stressful situations, are at the same time associated to damaging behavior outbreaks (50, 107). Similarly, resting can be used to extrapolate maternal ability of sows, as it is associated to nursing behavior (37), and thermal comfort in the pen (34). Lying posture can also be used for assessing diurnal activity patterns of the animals (79). Image technologies that detect locomotion and axial body-movement are promising tools for assessing lameness, an important welfare issue (113), especially in sows (35).

Image based technologies are also able to accurately assess drinking behavior and water usage, which are acknowledged to be crucial for pig welfare (73, 74). Vision-based technologies have great potential of assessing animal welfare by continuously monitoring behaviors of pigs, which can be used to detect changes and deviations in normal behavioral patterns related to animals' affective state (150). Some specific features such as the posture of the tail can provide useful information in relation to tail biting outbreaks or can even be related to the affective state of the pig (150). Besides, computer vision can provide information on behavioral changes such as interactions between individuals, allowing the detection of aggressive events and affiliative behaviors as nursing and playing (37). The use of image-analysis to evaluate the cascade defense has been validated in just one study, however, it still shows the potential of this tool to assess fear and stress-associated conditions (105).

Body weight detection, individual recognition, behavior and activity tracking are the most frequent uses of commercial image PLF technologies. According to Wurtz et al. (148), one of the difficulties of camera-based technologies is to monitor animals at individual level. Nevertheless, results on studies validating vision algorithms for individual identification and location, seem to be promising (84). Image-based individual recognition is not invasive, and can be used in real-time, helping to overcome some of the limitations of RFID systems (stress to the animals when attaching an RFID tag, and time requirements to the farmer in attaching and reading). Current protocols, such as Welfare Quality® (2), assess the nutritional state of animals by the body-condition. Image-analysis seems to be a promising tool to improve the assessment of the nutritional status continuously, by monitoring the body size (117, 122). Compiled results on camera-based systems in farm conditions for pig weight estimation, show potential of these tools for reducing the need of human-animal interaction, reducing stress associated to an unfamiliar human presence (118, 119, 124). Besides, camera-based PLF allows to monitor specific situations as farrowing, and the detection of the number of piglets in the farrowing pen, which has been studied to prevent perinatal asphyxia and piglets' crushing (83).

#### Load Cells and Flow Meters

Flow meters are discussed in the same section with load-cells, as its application for welfare assessment is strongly related to monitoring of feed-intake. Load-cells also include force plates.

Scales without individual identification have been used for body weight measurement. Reported deviations are around 1 kg at group (39) and individual level (123). In combination with RFID, load-cells systems (electronic feeding stations), could estimate body weight with a percentage error of 3% (52), showing less accuracy than an ordinary scale. Monitoring the feed intake by measuring the feed weight in an electronic station with RFID was found to reach a 90% accuracy (85). An overestimation of 1.1% of feed intake has been found in one study (49). Feeding patterns (time and frequency) of individual growing-finishing pigs can be analyzed by combining RFID and load-cells, reaching an accuracy of 97% (58, 86). RFID data for measuring the drinking behavior of individual pigs, showed 93% of accuracy (93).

Load-cell technologies allow to monitor body weight and growing patterns at group level. When working with RFID, load-cells can monitor feeding and drinking patterns and growing performance at individual level, overcoming one of the challenges that cannot be achieved by current welfare protocols, which can only monitor these aspects at group level. Although a normal growth pattern may have little predictive value in terms of good animal welfare, growth deviations or retardations have been used to identify health issues and other welfare problems (137). Automatic feeders with RFID are a promising technique to understand animals' requirements and anticipate welfare problems based on feeding patterns deviations, allowing the implementation of corrective measures and thus improving animal health and welfare (46).

##### Force Plates

Lameness is a frequent and important welfare problem, because of the intense pain it causes, the disadvantages that it brings in terms of access to food and water (151, 152). Also, in the normal housing conditions of a pig farm, which mainly use slatted floor (151), may only exacerbate the problem. Due to stocking density, and subjectivity of observations, the usual visual diagnosis of lameness is challenging. The most affected animals often lose feeding times, and consequently body condition decreases, which gets the attention of farm staff, and that is when observation is usually performed. Early diagnosis of lameness can prevent the associated high culling and mortality rates, especially for sows (152). Force plates are accurate for evaluating gait characteristics and detecting lameness even at an early stage (40). Several validation studies confirm their potential (35, 40, 109–111, 153). Different features have been extracted and validated using visual observation as a gold standard (109, 110, 153). Weight distribution of legs (percentage of weight, ratio between the weights applied by contralateral legs, weight shifting, amplitude of weight bearing and weight removing) significantly correlated with the golden standard (CV = 5.22%) (111). Weight shifting frequency and the ratio between the weights applied by contralateral legs performed the best in terms of identifying lame individuals (109).

##### Flow Meters

The use of flow meters to assess drinking patterns and water usage, have proved useful for prediction of several welfare conditions, such as presence of disease (38), and tail biting outbreaks (108). Performance of warning algorithms based on deviations from expected diurnal pattern in water consumption, showed that the algorithms were capable to predict a diarrhea outbreak 1 day before presentation of clinical signs (38).

#### Accelerometers

Accelerometers have been used to classify postures and activity with a performance for detecting and classifying activity ranging from 75 to 100% (60–64, 77). By classifying postures and activity nest-building behavior can be monitored to predict farrowing time with an accuracy of 86% (98). Acceleration data have also been used to detect lameness based on sow postures with an accuracy of up to 93% (131). Acceleration in combination with body temperature data was tested for generating early alerts of disease, reaching 97% of sensitivity and 89% of specificity (44).

Deviations in activity pattern might point out to health issues (44) and lameness (131). Accelerometers can therefore provide useful information, but the application on pig farms will be limited because sensors have to be attached to individual pigs, implying handling stress. For instance, accelerometers may be embedded in ear tags, which requires the perforation of an animal's ear for placement. Another alternative is the attachment of the accelerometer on the animal's back or leg, but ensuring that the device remains in place can lead to complications. Besides, the maintenance of a device attached to the pigs' bodies could be difficult under farm conditions, as it can motivate other pigs' chewing behavior in response to novelty of an object (154). Short battery life of wearable sensors is also a limitation of its applicability on farm. However, optimization of power consumption and battery life are currently being improved (65). For lameness detection, accelerometers can be mainly relevant to be used in sows.

#### Microphones

Microphones accuracy for assessing and classifying vocalizations was >73% (eight out of nine validation trials studies). One sound-analysis algorithm reached an accuracy of 98% distinguishing stress vocalizations associated to pain, using duration and intensity of vocalization signal as a gold standard (91). The detection of vocalizations related to hunger, thirst, cold and heat conditions (ranged from 69 to 71%) (91). Cough detection for localization of sick pigs at barn level using microphones, reached an interval of confidence of 95% (135). It was also found an accuracy from 73 to 93% for correct identification ratio of sick pigs cough sounds (136).

Sound analysis has been used for detecting coughing pigs. Coughing is a sign of respiratory problems or at least of poor climate conditions (dust, ammonia). Measuring coughing is therefore a relevant indicator contributing to animal welfare assessment, although it cannot be done at the individual level. Furthermore, if stress and pain related vocalizations can be reliably identified, it could also be used to further welfare aspects such as stress assessment and fighting events, for instance. Distress vocalizations induced by hunger, or extreme thermal discomfort seem to be more difficult to classify than vocalizations due to pain (91). Future research is needed in a larger vocal spectrum of vocal signals, not only to assess negative welfare aspects but also for assessing positive welfare.

#### Thermal Cameras

Thermal cameras are mainly used for remote sensing of body temperature (17). Body temperature is relevant in relation to animal welfare because over certain thresholds it can evidence hyperthermia or hypothermia and may also reflect fever. Besides, thermal imaging seems to be a promising tool for monitoring physiological responses as inflammation related to lameness (132), and animals' distribution responding to housing thermal conditions (81). Additionally, thermal imaging can be a promising tool for assessing acute stress events by body temperature changes (89). Thermal image for predicting stress in piglets, reached accuracies of 50, 86, 91, and 100% when stress was related to pain, hunger, thirst, and cold, respectively (89). Thermal cameras for assessing animals' space distribution (clustering behavior) in function of body temperature and radiated temperature, was validated showing a significant correlation between clustering and temperature response (81). Also, the correlation between thermal image measurements and rectal temperature was high (*r* = 0.80) (126). Inflammation related to lameness in pregnant sows was also detected using thermal imaging, showing significant correlation between mean upper metatarsal temperature and sows' parity (132). Therefore, thermal imaging allowed to differentiate between lame and non-lame sows, and to detect temperature differences in the affected leg. Hence, the welfare problem resulting from the pain caused by the inflammation associated with lameness (151), can be detected by thermal imaging. Thermal imaging at chest level for the diagnosis of lung tissue alterations associated with *Actinobacillus pleuropneumoniae* infection, by measuring the body temperature at chest level, reached a specificity of 100% (134).

#### Photoelectric Sensors

Photoelectric sensors, the only sensor group with external validation records for activity measurements, showed a precision lower than 90% (56, 66). The potential of these sensors to detect position changes in puerperal sows showed 64% of sensitivity and 88% of specificity (59). For monitoring activity levels in pigs, photoelectric sensors detected movement in <1 s (67).

Photoelectric sensors can detect movement and therefore provide useful information about the activity level and postural transitions, which contribute to welfare assessment. According to Besteiro et al. (56), these sensors work better with recently weaned piglets and assessing play than feeding behavior. As the body-weight of animals increases, the coverage area of the photoelectric sensor decreases, resulting in less precise measurements. In contrast, the detection of intense activity is more precise than non-intense activity (56).

#### RFID

RFID technology used for individual recognition of multiple pigs at the same RFID reader in the pen, can reach an accuracy of 92% (41). It has been demonstrated that the use of two RFID tags instead of one, increased the accuracy up to 97% (58, 86). Deviations in feeding patterns as an indicator of disease, monitored by RFID data, have showed an accuracy of 97%, and a precision of 71% (137).

RFID is used for individual identification, and this is essential if we want to opt for an increasingly individualized welfare evaluation. RFID is very useful in combination with many other devices such as scales and automatic feeders and drinkers. RFID allows to track animals' location. It may offer additional practical applications such as monitoring social interaction as a possible transmission path for diseases (155), as the contact intensity and length between individuals may be an indicator for disease transmission (156).

#### Non-contact Body-Temperature Sensors

Non-contact body-temperature sensors revealed its limited usefulness as an alternative to sensing temperature measurement. The one study found on pyrometer for continuously measuring pigs' body temperature showed that the performance was not reliable (55). Under fever-induced situations, comparing vaginal thermometer data and pyrometer data in the orbital area of animals in time periods from 0.25 to 5 h, a positive correlation was found only in a third of the sample. The longer the measuring period, the fewer animals showed a significant correlation. Similar conclusions were obtained from testing the accuracy of infrared thermometers for body-temperature measurement, compared to rectal temperature as a gold standard (133). Several authors conclude that environmental conditions such as ambient temperature, sunlight, air movement, barn and pen configuration, and stocking density, have a significant impact on the reliability of infrared thermometry to assess body-temperature in pigs (133, 157).

##### Trends and Gaps in PLF Technologies for Pig Welfare Assessment

To increase transparency of animal production, there is a need for reliable data on the welfare of farmed animals. This information is above all important for the animals as if used in a proper way it can improve their lives. It can also assist both consumers and producers to make decisions from an informed perspective. For the sake of the animals and production efficiency, producers need to monitor the health and welfare status of animals. It may be done with reliable and up-to-date information as early-warning systems, before implementing corrective and timely measures. Consumers are demanding clear information about farm animal welfare to assist them in identifying and choosing enhanced welfare-friendly products. Recent advances in sensor technologies increasingly allow systematic and automated monitoring of several indicators that inform about the welfare status of farm animals. This data could be transformed to useful information for consumers as labeling.

However, there is a need to identify and select the most appropriate indicators and the relevant PLF technologies to assess them. This review, is the first of its kind, spotting relevant technologies that can assist on this task. Nevertheless, we identified some challenges and gaps that need to be addressed. To date, welfare has been based on focal assessments, and as information is mainly applicable to the day in which the evaluation is carried out, a limited picture of welfare status of animals is provided. PLF technologies dispense continuous welfare information using both behavioral (e.g., activity) and physiological indicators (e.g., body temperature and weight), which could yield a continuous and systematic assessment at different stages of their life, and in the future, may revolutionize the way animal welfare is evaluated. This may allow to investigate deviations from normality at the individual level, leading to one welfare appraisal which is predominantly animal-based, and that is less dependent on environmental-based indicators. Deviations from “normal” patterns at individual level will account for individual differences rather than trying to understand “average” animals. Information on the evolution of animal behavior and welfare throughout an animal's lifetime and throughout the chain may facilitate understanding of factors impairing or promoting it. This understanding of animal welfare will further be reinforced by accessibility to large data sets, only available with the integrated automatic and systematic assessment.

There is thus a need for an integration of the different aspects of animal welfare (i.e., health, nutrition, comfort, affective state and natural behavior) into relevant information that could assist stakeholders to make decisions. The combination of sensors may provide more relevant information than if taken separately as animal-based indicators can be related. For instance, the use of one activity sensor may alert when an animal stops moving, which could be a sign of different health problems (e.g., lameness, disease), but if the activity sensor is combined with a thermal camera informing about body temperature, the welfare information delivered can be much more precise. In order to cover these needs, block chain technology has been judged useful to integrate information throughout the entire production chain and monitor welfare at different stages of the animal's lives (158, 159).

Market availability and validation records of sensor technologies dedicated to animal-based welfare monitoring in dairy industry has been recently conducted (28). There are clear differences between dairy and pig industry when it comes to market availability, type of sensors used and validation records. It seems that the pig industry is behind dairy regarding sensor availability (and validation), especially when population numbers are compared [pigs around 677.6 millions of heads (160) against to 270 million oh head in dairy cattle (161)]. Looking on the nature of production, pigs are mostly kept in groups, and very often are not individually identified. Since lifespan of a productive pig is limited (excluding sows), individual identification is relatively expensive and more difficult to manage (145, 162). Nevertheless, individual identification allows for a more specific picture of any sub-optimal state of well-being, which is not captured by group averages (46, 58, 85, 86, 93, 145). There is a difference in the investment on individual animals' identification in function of their productive objectives. Sows are more commonly identified by RFID tags than fattening pigs, especially in farms using electronic feeding stations, as their productive lifespan is longer. In fattening pigs, group monitoring is more common as it reduces the costs of assessment. This might be a reason why some technologies, which would have great potential for health and welfare monitoring, are so scarcely represented on the market.

Based on market analyses, it is clear that availability of vision-based monitoring for pigs are greater than in cattle production. It could be due to cost concerns (163). For example, in order to monitor the body weight of fattening pigs, few technologies could be considered. Using a weight sorting system based on load cells and RFID requires substantial investment on farms and might only be feasible in newly constructed farms (164), while cameras can be installed also to already operating systems with potentially less financial input. Interestingly, neither of those systems are validated externally.

In conclusion, existing PLF technologies are potential tools for on-farm animal welfare assessment in pig production. A variety of animal-based welfare indicators can be monitored on an individual scale, continuously and in real time, using PLF. These tools had demonstrated potential for yielding a continuous and systematic assessment at different stages of animals' lives, overcoming some difficulties and gaps of current welfare assessment protocols. Thus, in the future, PLF may revolutionize the way animal welfare is assessed and informed. However, validation studies are lacking for an important percentage of market available solutions, and in particular, research and development need to focus on identifying feature candidates of the measures (e.g., deviations from diurnal pattern, threshold levels etc.) that are valid signals of either negative or positive animal welfare. An important gap identified are the lack of technologies to assess affective states (both positive and negative states).

In this review, tools were validated against three possible golden standards: human observer, other tool with well-defined performance record, or based on the tool's ability to detect change in animal behavior or physical condition during planned experiment. The need for an established protocol for the validation procedures of PLF technologies can be noticed, as the measurements presented in the performance reports are very heterogeneous.

## Data Availability Statement

The original contributions presented in the study are included in the article/Supplementary Material, further inquiries can be directed to the corresponding author/s.

## Author's Note

This study is conducted within ClearFarm project which aim is to co-design, develop, and validate a software platform powered by the integration of PLF technologies, to assess animal welfare throughout the production chain, and to provide this information to all parts of it.

## Author Contributions

YG, AS, and PL conceived the idea of this manuscript. YG defined and performed the literature search and the company search. YG and AS conducted the literature screening. YG wrote the paper with input from AS, PL, JN, MP, EB, IB, LP, and XM. All authors contributed to the article and approved the submitted version.

## Conflict of Interest

The authors declare that the research was conducted in the absence of any commercial or financial relationships that could be construed as a potential conflict of interest.
